# Editorial: Promoting mental health and wellbeing among young people

**DOI:** 10.3389/fpubh.2025.1706563

**Published:** 2025-09-29

**Authors:** Sigurd Lauridsen, Susan Andersen, Maj Britt Dahl Nielsen

**Affiliations:** The National Institute of Public Health, University of Southern Denmark, Copenhagen, Denmark

**Keywords:** mental health, anxiety, depression, COVID-19, school climate, online behavior

Globally, more than one in 10 children and adolescents live with a diagnosable mental disorder, such as anxiety, depression, or self-harm, highlighting the urgent need for coordinated action (1). While prevalence rates vary across regions, the overall trend points to a growing global mental health crisis among youth (2).

This Research Topic responds to that crisis by examining trends, determinants, and barriers to mental health care, while showcasing effective, scalable, and culturally responsive interventions. Featuring 41 contributions from Asia, Europe, America, and Oceania, this Research Topic reflects a wide geographical and cultural scope. The Research Topic spans diverse methodologies, including longitudinal studies, cross-sectorial studies, randomized trials, systematic reviews, and qualitative research, offering a comprehensive overview of both the challenges facing today's adolescents and young adults, and the solutions being developed to support their mental health and wellbeing.

Although the themes covered are wide-ranging, eight interconnected transversal themes emerge: school climate; COVID-19; online behaviors; community, culture, and environment; mental health literacy, stigma and access to care, and promoting mental health. Together, these themes illustrate both the shared global challenges and the context-specific drivers of youth mental health, while also pointing toward possible solutions. For an overview of the themes (see Figure 1).

**Figure 1:**
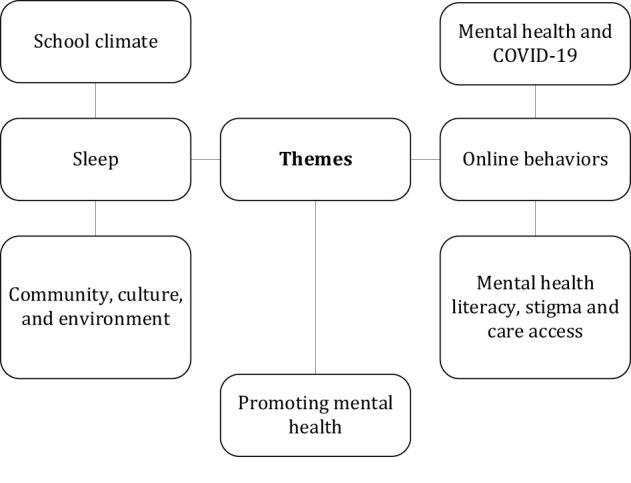
Themes in the Research Topic: Promoting mental health and wellbeing among young people.

## School climate

Studies in this Research Topic highlight how adolescent mental health reflects the interaction of socioeconomic resources, school environments, family dynamics, and cognitive-emotional processes. Most of the studies are by Chinese authors, reflecting the impact of a very competitive educational sector on the mental health of young people.

Studies in this theme show that financial resources protect against distress, while hardship exacerbates vulnerability as demonstrated in both a study among Ghanaian university students (Quansah et al.), and in a scoping review mapping the evidence on the prevalence of and associated risk factors of stress among adolescents in China (Hao et al.). Girls and older students reported higher stress levels (Ma et al.) and students experiencing study stress or studying medicine or health sciences reported higher levels of depressive symptoms. Furthermore, rural freshmen exhibited significantly higher levels of anxiety and depression than their urban counterparts (Li and Sun). While academic anxiety was common among adolescents facing college entrance exams, network analyses showed that somatic complaints, social anxiety, and self-blame were central factors (Fang et al.).

School belonging emerged as a buffer against depressive symptoms (Yin et al.), while maladaptive patterns, such as rumination, was negatively related to quality of life (Yu and Zhao) and post-stress growth (Wang Z. et al.). A mediation analysis indicated that higher family income and parental literacy were associated with elevated levels of academic buoyancy in children, i.e., students' ability to deal with the frustrations and challenges of school daily. Conversely, mental health problems showed a negative relationship with academic buoyancy (Hu et al.). Moving beyond academic stressors, Bonsaksen et al. revealed that about a third of Norwegian junior high school experiences sexual harassment, with girls more likely to be affected. Exposure was linked to more depressive symptoms, greater loneliness, and lower self-esteem and wellbeing.

## Mental health and COVID-19

The COVID-19 pandemic intensified mental health risks among youth, with studies in this theme highlighting the role of social disconnection, trauma, and inequality. Moreover, the studies also demonstrate how the pandemic affected youth in diverse ways across different contexts.

Several studies focused on the impact of disrupted peer contact and changing social dynamics. Fu H. et al. and Pinkse-Schepers et al. found that restricted peer interaction in China and the Netherlands was associated with increased depression and social anxiety—especially among girls. In a qualitative study, Sobotka et al. demonstrated that challenges persisted during the reopening phase, as the return to in-person schooling introduced new stressors even after pandemic concerns had faded.

Importantly, not all young people were affected equally. In Saudi Arabia, Alasqah et al. found that female university students experienced higher levels of stress, depression, and anxiety symptoms. Sun et al. examined a particularly vulnerable group—nursing interns in China—and reported high levels of PTSD following the liberalization of COVID-19 control policies. Key risk factors included direct care of infected patients and fear of contagion.

Other research examined psychological mechanisms alongside broader social and contextual conditions. Fu C. et al. found that intolerance of uncertainty was positively associated with negative emotions, while psychological capital and family support served as protective factors among Chinese students. Broader contextual influences were also evident: Mollaesmaeili et al. reported that perceptions of urban green spaces in Iran were linked to fear of COVID-19 recurrence, anxiety, and depression, whereas Kim et al. found that leisure satisfaction in Korea was associated with lower perceived infection risk. Collectively, these studies demonstrate that resilience during crises depends not only on individual coping mechanisms, but also supportive social, family, and environmental resources.

## Sleep

Sleep emerges as a transdiagnostic factor in youth mental health. Although few contributions focus specifically on sleep, the findings underscore its relevance across diverse aspects of mental health, including mood regulation and suicidality. Zhang X. et al. found that poor sleep quality and shorter sleep duration were associated with higher depressive symptoms among Chinese adolescent, while Slanitz et al. reported a link between poor sleep and suicidality in Austrian young adults, mediated by anxiety and depression. While the number of studies abut sleep is limited and cross sectional, they point to sleep as a central component of youth mental health intersection with emotional, cognitive, and behavioral outcomes.

## Online behaviors

Online behaviors play a central role in young people's lives, creating opportunities for connection and learning but also exposing them to new risks. Studies in this theme examine problematic patterns such as harassment, nomophobia, and Internet addiction. Nielsen M. B. D. et al. revealed widespread online harassment and bullying among Danish high school students, with notable gender differences. Ceobanu et al. examined psychological factors underlying nomophobia—the fear of not being in possession of your mobile phone or losing your mobile phone—among Romanian university students. They found that fear of missing out, rumination, and non-pathological compulsions were key contributors. Zhang J. et al. identified distinct joint trajectories of Internet addiction and depressive symptoms among Chinese adolescents, and found that individual, family, and school factors predicted membership in these trajectories. Collectively, these studies illustrate how online behaviors interact with offline vulnerabilities, underscoring the need for further research.

## Community, culture, and environment

This theme includes studies grouped under the broader category of community, culture, and environment, focusing on how these contextual factors shape youth mental health. Some contributions focus specifically on the environmental dimension. Grande et al. adopted a participatory approach with Indigenous students in Brazil to identify priorities for supporting mental health during climate change crises, while Wang J. et al. examined how urban green spaces can promote wellbeing among young adults.

The role of community and cultural influences is explored in several other studies. Battistella-Lima and Veludo-de-Oliveira investigated the protective effects of gratitude against materialism, Wiium et al. examined the resilience-building potential of civic engagement, and Brockie et al. presented a protocol for studying suicide clusters among Native American youth, linking them to historical trauma.

A common feature across several studies is the active involvement of young people in the research process, ensuring that their perspectives and lived experiences are meaningfully represented. Ow et al. complement these context-focused studies by highlighting how young people themselves conceptualize health as embedded in their social and environmental surroundings, underscoring the importance of including youth perspectives in the development and evaluation of mental health services. Together, these studies reflect the diverse ways in which community, cultural identity, and environmental factors intersect with youth mental health across different settings.

## Mental health literacy, stigma and access

Young people's willingness to seek help for mental health challenges is shaped not only by what they know, but also by how they feel about mental illness and the systems meant to support them. Studies in this theme examine how mental health literacy, stigma, and structural barriers influence help-seeking behavior. Several contributions focus on the role of mental health literacy among young people and their caregivers. Kusaka et al. investigated mental health literacy among Japanese caregivers of teenagers, finding that while most were willing to support teens experiencing mental health problems, their actual knowledge about mental health was limited. Sokolová reported that Slovak students with higher mental health literacy showed lower self-stigma related to seeking professional help, with notable differences based on exposure to psychology education.

Beyond literacy, stigma and access to care are also central concerns. Joulaei et al. documented substantial unmet mental health care needs among Iranian adolescents, with common barriers including reluctance to seek help and difficulties accessing services. Hyseni Duraku et al. used a mixed-methods design to investigate help-seeking barriers among university students in Kosovo, revealing a lack of trust in mental health professionals and difficulties in self-disclosure.

Taken together, these studies illustrate that knowledge alone does not guarantee service use; trust, stigma, and social context remain critical factors shaping pathways to care.

## Promoting mental health

This theme highlights a range of strategies for promoting youth mental health across everyday settings. Two contributions shed light on community-based approaches: Andersen et al. showed that peer self-management may reduce depression and anxiety, while Tuaf and Orkibi presented a scoping review of 27 programs targeting adolescents with mental health challenges.

Several studies explored health-promoting interventions in university settings. Nielsen L. et al. proposed a multi-level framework for mental health promotion in higher education; Brown et al. reported findings from a pilot study of an exercise-based program; and Wang F. et al. offered qualitative insights from an initiative that trained mental health providers to become campus-based champions. In addition, Zuo et al. conducted a meta-review of 11 randomized controlled trials (RCTs), showing that mindfulness interventions among college students can reduce depression and anxiety and improve sleep, though effects on mindfulness scores were limited.

These contributions demonstrate how youth mental health promotion can take many forms, like peer support, mindfulness, empowerment, and recovery-oriented programs, adapted to diverse cultural and institutional contexts and grounded in inclusion and active engagement.

## Future directions

The eight themes in this Research Topic reveal a consistent pattern: youth mental health is affected by a dynamic interplay of individual vulnerabilities, relational support, institutional contexts, and broader societal forces. Insights which cohere with other studies of the mental health of young people that points to a rise in poor mental health especially in terms of depression and anxiety (3–6).

Future research must prioritize multilevel, longitudinal, and cross-cultural approaches that capture these complex dynamics.

Policy and practice should focus on six interconnected areas that reflect the evidence presented in this Research Topic, from everyday settings to emerging digital landscapes:

1. Embedding mental health promotion across schools and universities to address mental health where young live their daily life.
2. Integrating family and community contexts into interventions, recognizing that resilience is socially embedded.
3. Expanding prevention to target sleep, mental health literacy, stigma, and early recognition of symptoms and easy access to health care.
4. Addressing socioeconomic inequalities, ensuring that financial and structural supports reduce vulnerability.
5. Preparing for future crises, whether pandemics or climate change by investing in sustainable support systems.
6. Addressing the mental health impact of digital environments and emerging technologies, including AI. While not central in this Research Topic, AI is rapidly entering youth mental health—offering new solutions and treatments but also raising ethical concerns.

Ultimately, promoting youth mental health is both a clinical and societal responsibility. The evidence gathered here underscores that meaningful progress depends on coordinated action across education, health, community, and policy sectors.
